# Supplementary material for: In Situ Construction of CNT/CuS Hybrids and Their Application in Photodegradation for Removing Organic Dyes
Source: Nanomaterials (Basel). 2020 Jan 20;10(1):178. doi: 10.3390/nano10010178 (PMC7022525; doi:10.3390/nano10010178)
Supplement: Supplementary file 1 [file nanomaterials-10-00178-s001.pdf]

Supporting Information

In-situ construction of CNT/CuS hybrids and their promising photodegradation application for removing organic dyes

Yanping Wang [1], Fuchuan Jiang [1], Jiafu Chen [2], Xiaofeng Sun [3], Tao Xian [3] and Hua Yang [1,*]

[1] School of Science, Lanzhou University of Technology, Lanzhou 730050, China; wangyanpinglut@163.com (Y.W.); jiangfc_sir88@163.com (F.J.)
[2] Ministry of Education Key Laboratory of Testing Technology for Manufacturing Process, Southwest University of Science and Technology, Mianyang 621010, China; flyingbanana@yeah.net
[3] College of Physics and Electronic Information Engineering, Qinghai Normal University, Xining 810008, China; sunxf027@126.com (X.S.); 2016105@qhnu.edu.cn (T.X.)
* Correspondence: hyang@lut.cn; Tel.: +86-931-297-378-3

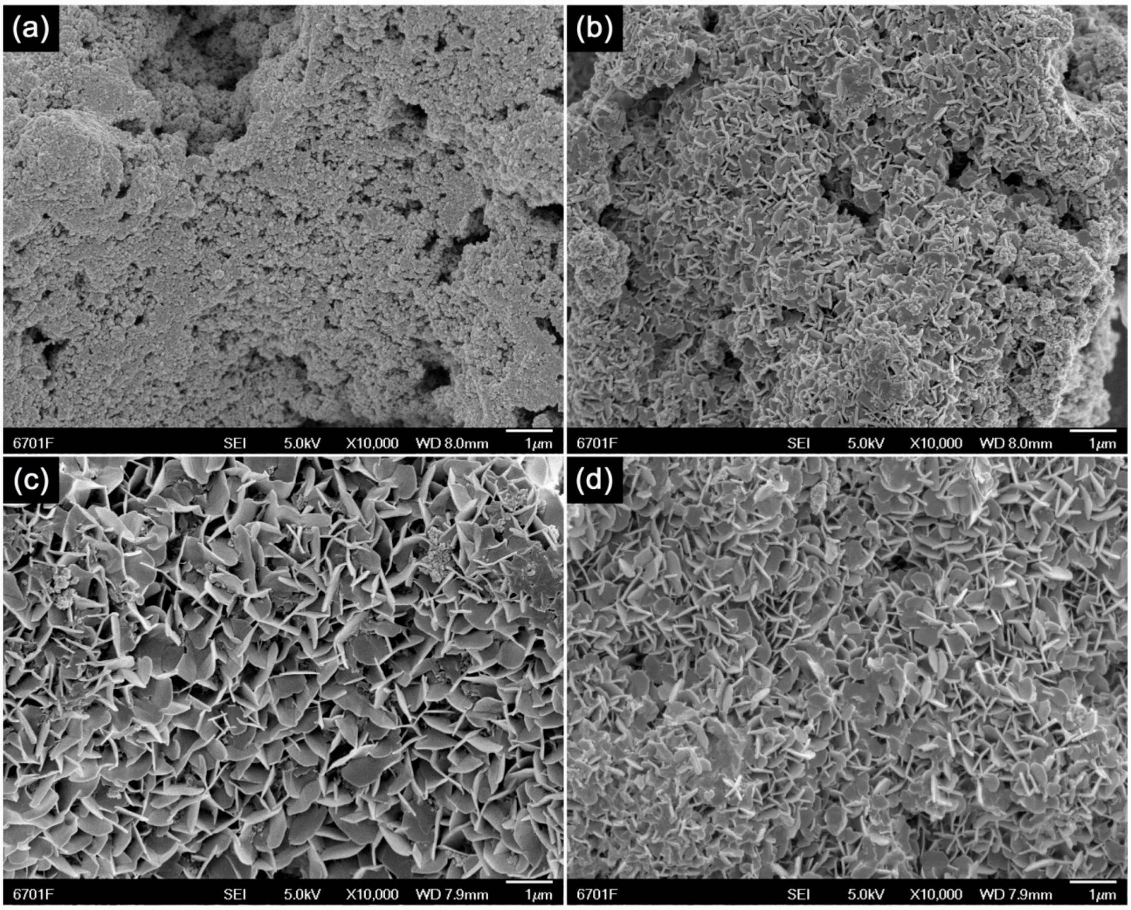

Figure S1. SEM images of CuS samples synthesized at 20 ^{o}C with different reaction times. (a) 0.5h-20°C, (b) 2h-20°C, (c) 10h-20°C, and (d) 12h-20°C.

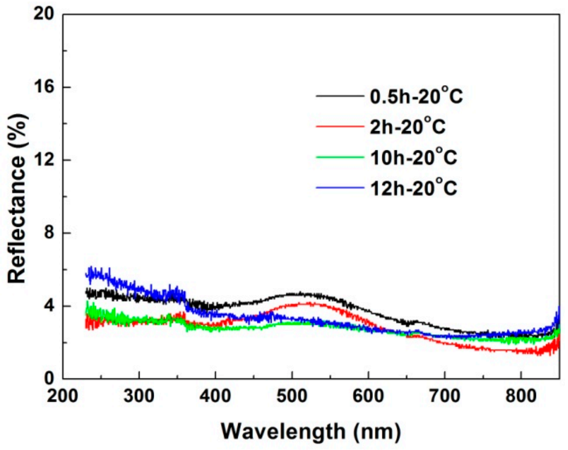

Figure S2. Ultraviolet-visible diffuse reflectance spectroscopy (UV-vis DRS) spectra of CuS samples synthesized at 20 °C with different reaction times.

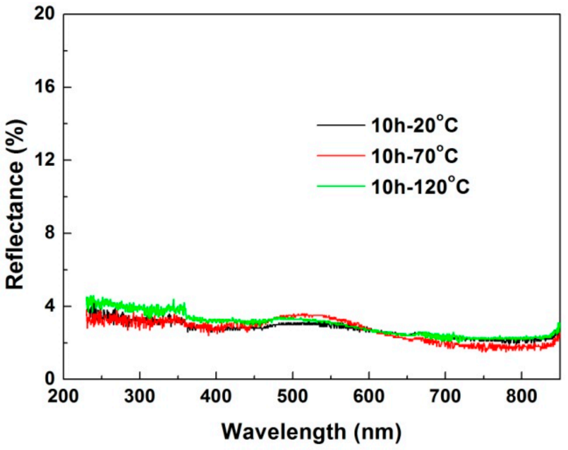

Figure S3. UV-vis DRS spectra of CuS samples synthesized at different reaction temperatures for 10 h.

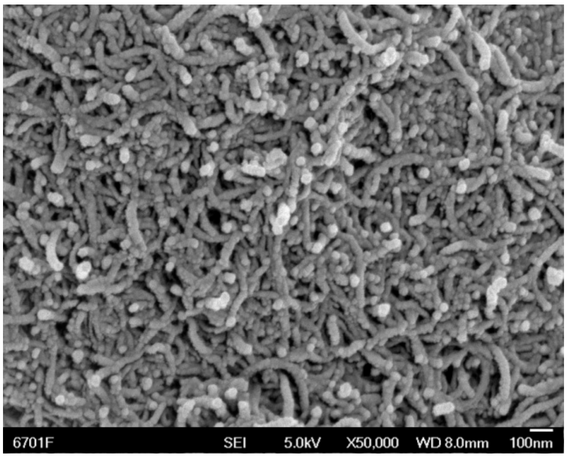

Figure S4. SEM image of pure CNTs.

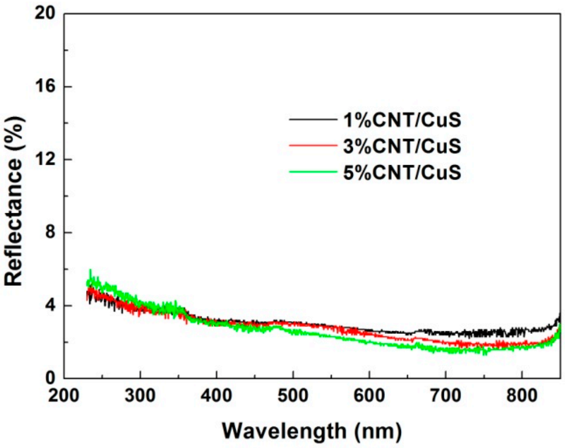

Figure S5. UV-vis DRS spectra of the CNT/CuS composites.

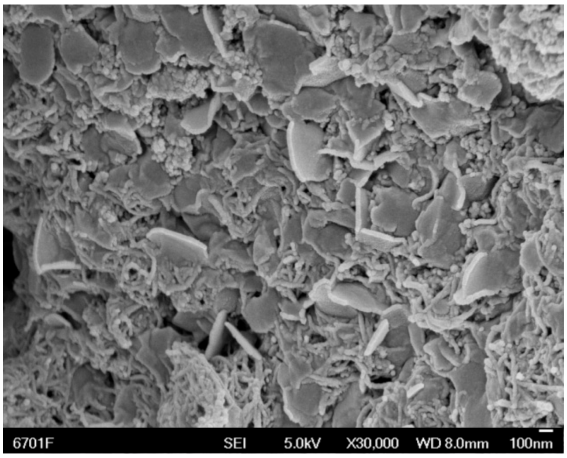

Figure S6. SEM image of the 3%CNT/CuS-MD composite obtained by mechanically mixing CuS nanoflakes and CNTs and then drying.
